# Recommendation of Antimicrobial Dosing Optimization During Continuous Renal Replacement Therapy

**DOI:** 10.3389/fphar.2020.00786

**Published:** 2020-05-29

**Authors:** Lu Li, Xin Li, Yanzhe Xia, Yanqi Chu, Haili Zhong, Jia Li, Pei Liang, Yishan Bu, Rui Zhao, Yun Liao, Ping Yang, Xiaoyang Lu, Saiping Jiang

**Affiliations:** ^1^Department of Pharmacy, College of Medicine, The First Affiliated Hospital, Zhejiang University, Hangzhou, China; ^2^Department of Pharmacy, Second Hospital of Jilin University, Changchun, China; ^3^Department of Pharmacy, The First Affiliated Hospital, Sun Yat-sen University, Guangzhou, China; ^4^Department of Pharmacy, Xuanwu Hospital of Capital Medical University, Beijing, China; ^5^Department of Pharmacy, First Affiliated Hospital of Nanchang University, Nanchang, China; ^6^Department of Pharmacy, Nanjing Drum Tower Hospital, Nanjing, China; ^7^Department of Pharmacy, Tianjin First Central Hospital, Tianjin, China; ^8^School of Medicine, Sir Run Run Shaw Hospital, Zhejiang University, Hangzhou, China; ^9^Department of Pharmacy, Tongren Hospital, Shanghai Jiao Tong University School of Medicine, Shanghai, China

**Keywords:** antimicrobials, continuous renal replacement therapy, dosing optimization, pharmacokinetic, pharmacodynamics

## Abstract

Continuous Renal Replacement Therapy (CRRT) is more and more widely used in patients for various indications recent years. It is still intricate for clinicians to decide a suitable empiric antimicrobial dosing for patients receiving CRRT. Inappropriate doses of antimicrobial agents may lead to treatment failure or drug resistance of pathogens. CRRT factors, patient individual conditions and drug pharmacokinetics/pharmacodynamics are the main elements effecting the antimicrobial dosing adjustment. With the development of CRRT techniques, some antimicrobial dosing recommendations in earlier studies were no longer appropriate for clinical use now. Here, we reviewed the literatures involving in new progresses of antimicrobial dosages, and complied the updated empirical dosing strategies based on CRRT modalities and effluent flow rates. The following antimicrobial agents were included for review: flucloxacillin, piperacillin/tazobactam, ceftriaxone, ceftazidime/avibactam, cefepime, ceftolozane/tazobactam, sulbactam, meropenem, imipenem, panipenem, biapenem, ertapenem, doripenem, amikacin, ciprofloxacin, levofloxacin, moxifloxacin, clindamycin, azithromycin, tigecycline, polymyxin B, colistin, vancomycin, teicoplanin, linezolid, daptomycin, sulfamethoxazole/trimethoprim, fluconazole, voriconazole, posaconzole, caspofungin, micafungin, amphotericin B, acyclovir, ganciclovir, oseltamivir, and peramivir.

## Introduction

Infection is the one of the main causes of acute kidney injury (AKI), and renal replacement therapy is necessary for such patients. Sepsis causes more than half of all AKI (Bellomo et al., 2017), and therefore critically ill patients who receive renal replacement therapy often need to use antimicrobials at the same times. Continuous Renal Replacement Therapy (CRRT) is a therapy that replaces the normal blood-filtering function of the kidneys which continues for at least 24 h. Compared with intermittent dialysis, CRRT is characterized by hemodynamic stability during treatment, and can maintain the fluid, electrolyte and acid alkaline balance in efficient and smooth manners (Deepa and Muralidhar, 2012). With the increasing use of CRRT, it is particularly important to understand the impact of CRRT on antimicrobial therapy (Bouchard et al., 2008). For patients using CRRT, there are many factors that may affect the antimicrobial pharmacokinetics (PK)/pharmacodynamics (PD), such as CRRT factors, patient individual differences, drug characteristics, and so on (Pea et al., 2007; Roberts et al., 2020). Inappropriate doses of antimicrobial agents may lead to increased adverse drug reactions, pathogen resistance, and clinical treatment failure. With the improvement of CRRT equipment, techniques, prescriptions (Macedo and Mehta, 2016), and as the PK/PD studies go on, previous studies do not reflect contemporary CRRT techniques (Trotman et al., 2005; Seyler et al., 2011) and prescriptions (Shaw and Mueller, 2017; Pistolesi et al., 2019) or include only a small number of agents (Hoff et al., 2019).

In the present work, we reviewed the studies and summarized the pharmacokinetic parameters of common antimicrobials, including antibacterial, antifungal and antiviral agents (**Table 1**). We classified these drugs (**Table 2**) and updated the recommendations for dose adjustment of antimicrobials for adults (**Table 3**) from the literatures (the majority of which were published from 2010 to March 2020) under different CRRT modalities and flow rates.

**Table 1:** Pharmacokinetic parameters of healthy volunteers.

Antimicrobials	MW	Protein binding (%)	Vd	T_1/2_(h)	PK/PD characteristic	PK/PD target	excretion
**Flucloxacillin**	453	95	16.792 L	0.5–1.1	time dependent	%T > MIC	mainly through the kidney
**Piperacillin**	517	16–48	0.24 L/kg	1	time dependent	%T > MIC	mainly through the kidney
**Tazobactam**	300	–	0.4 L/kg	1	–	–	mainly through the kidney
**Ceftazidime**	636	<10	0.24 L/kg	1.9	time dependent	%T > MIC	mainly through the kidney
**Avibactam**	265	–	0.31 L/kg	2.7	–	–	mainly through the kidney
**Ceftriaxone**	554	85–95	5.8–13.5 L	8	time dependent	%T > MIC	through kidney and biliary
**Cefepime**	571	20	18 L	2.0	time dependent	%T > MIC	mainly through the kidney
**Ceftolozane**	667	16–21	13.5 L	3.1	time dependent	%T > MIC	kidney
**Sulbactam**	255	38	0.3 L/kg	1	time dependent	%T > MIC	mainly through the kidney
**Meropenem**	437.5	2	0.29 L/kg	1	time dependent	%T > MIC	mainly through the kidney
**Imipenem**	240	15–25	0.27 L/kg	1	time dependent	%T > MIC	mainly through the kidney
**Panipenem**	339	6–7	10.2 ± 0.9 L	1	time dependent	%T > MIC	kidney
**Biapenem**	350	3.7	11.75 ± 3.86 L	1	time dependent	%T > MIC	kidney
**Ertapenem**	475	95	0.12 L	4	time dependent	%T > MIC	mainly through the kidney
**Doripenem**	420	8.1	16.8 L	1	time dependent	%T > MIC	mainly through the kidney
**Amikacin**	585	0–10	0.26 L/kg	2–3	concentration dependent	C_max_/MIC	mainly through the kidney
**Ciprofloxacin**	331	20–40	2.4 L/kg	4	concentration dependent	AUC_24_/MIC	mainly through the kidney
**Levofloxacin**	361	24–38	1.36 L/kg	7	concentration dependent	AUC_24_/MIC	mainly through the kidney
**Moxifloxacin**	401	30–50	2.2 L/kg	10–14	concentration dependent	AUC_24_/MIC	excreted by urine and feces
**Clindamycin**	425	85–94	1.1 L/kg	2.4	concentration dependent	AUC_24_/MIC	mainly excreted by biliary
**Azithromycin**	748	7–51	33.3 L/kg	68	time dependent, long PAE	AUC_24_/MIC	mainly excreted by biliary
**Tigecycline**	585	71–89	7–9 L/kg	42	time and concentration dependent	AUC_24_/MIC	excreted by urine and feces
**Polymyxin B**	1,189	60	0.07–0.2 L/kg	6	concentration dependent,	AUC_24_/MIC	mainly through non-renal ways
**Colistin**	1,748	>50	0.34 L/kg	2–3	concentration dependent	AUC_24_/MIC	mainly through the kidney
**Vancomycin**	1,448	10–55	0.7 L/kg	4–6	time and concentration dependent	AUC_24_/MIC	mainly through the kidney
**Teicoplanin**	1,709	90–95	0.9–1.6 L/kg	70–100	time dependent, long PAE	AUC_24_/MIC	mainly through the kidney
**Linezolid**	337	31	40–50 L	5	time dependent, long PAE	AUC_24_/MIC	65% is cleared by non-renal ways
**Daptomycin**	1,620	92	0.1 L/kg	8–9	concentration dependent	AUC_24_/MIC	mainly through the kidney
**SMX/TMP**	290	SMX:70 TMP:44	SMX:12–18 L; TMP:100–120 L	TMP:11 SMX:9	the pharmacodynamic parameter (concentration or time) has not been determined (Brown, 2014)	–	mainly through the kidney
**Fluconazole**	306	10	50 L	20–50	concentration dependent	AUC_24_/MIC	mainly through the kidney
**Voriconazole**	349	58	4.6 L/kg	Nonlinear pharmacokinetics	time and concentration dependent	AUC_24_/MIC	Less than 2% drug is eliminated by kidney as unchanged drug
**Posaconzole**	700	98–99	226~295L	20–66	time and concentration dependent	AUC_24_/MIC	mainly through the feces
**Caspofungin**	1,092	97	9.7 L	13	concentration dependent	AUC_24_/MIC	metabolites were excreted by urine (41%) and feces (35%)
**Micafungin**	1,270	>99	0.39 L/kg	14.0–17.2h	concentration dependent	AUC_24_/MIC	mainly through the feces
**AMB**	924		AMB: 4 L/kg; AMB lipid complexes:0.1–0.4 L/kg	AMB: 24; AMB lipid complexes: 6.8	concentration dependent	C_max_/MIC	Slowly excreted through the kidney, about 2–5% excreted every day
**Acyclovir**	225	9–33	0.7 L/kg	2.5–3.5	–	–	mainly through the kidney
**Ganciclovir**	255	1–2	0.7 L/kg	3.5 h	–	–	mainly through the kidney
**Oseltamivir**	312	3	23–26 L/kg	6–10	–	–	mainly through the kidney
**Peramivir**	346	<30	12.56 L	20	–	–	mainly through the kidney

MW, Molecular weight; MIC, minimum inhibitory concentration; AUC, Area Under Curve.

**Table 2:** Category of drugs in CRRT.

Category	Antimicrobials
**Drugs need adjustments**	flucloxacillin, piperacillin/tazobactam, ceftazidime/avibactam, ceftolozane/tazobactam, sulbactam, cefepime, meropenem, imipenem, panipenan, biaphenan, ertapenem, doripenem, amikacin, levofloxacin, ciprofloxacin, colistin, vancomycin, teicolanin, daptomycin, SMX/TMP, fluconazole, acyclovir, ganciclovir, oseltamivir, peramivir
**Drugs do not need adjustments**	ceftriaxone, moxifloxacin, clindamycin, azithromycin, tigecycline, polymyxin B, linezolid, voriconazole, posaconazole, caspofungin, micafungin, AMB

**Table 3:** Recommendation of drug adjustment in CRRT.

Drug	CRRT mode		
	CVVH	CVVHD	CVVHDF
**Flucloxacillin**	Ultrafiltration rate: 57 ± 9 ml/min 4.0 g q8h (Meyer et al., 2003)	–	–
**Piperacillin/tazobactam**	Ultrafiltration rate: 30 ml/kg/h Loading dose:4.5 g Maintenance dose: 500 mg/h (Roger et al., 2017a) Ultrafiltration rate: 1,000 ml/h to 2,150 ml/h Dose: 4.5 g q6h (Asin-Prieto et al., 2014)	Dialysate rate: 23 ± 9 ml/kg/h 4.5 g q6h or higher (for less susceptible pathogens) (Seyler et al., 2011)	Ultrafiltration rate: 15 ml/kg/h Dialysate flow rate: 15 ml/kg/h Loading dose:4.5 g Maintenance dose:500 mg/h (Roger et al., 2017a) Ultrafiltration rate: 22 ± 12 ml/kg/h Dialysate rate: 23 ± 9 ml/kg/h 4.5 g q6h or higher (for less susceptible pathogens) (Seyler et al., 2011)
**Ceftriaxone**	No change	No change	No change
**Ceftazidime/Avibactam**	–	–	Ultrafiltration rate: 0.25 L/h Dialysate rate: 1.5 L/h 2.5 g q8h (Soukup et al., 2019)
**Cefepime**	Ultrafiltration rate: ≤1,000 ml/h 1 g q8h Ultrafiltration rate: ≥1,500 ml/h 2 g q8h or 1 g q6h (Carlier et al., 2015) Ultrafiltration rate: 30.1 ± 5.4 ml/kg/h 2 g q8h extended infusion (Philpott et al., 2019)	Dialysate rate: 20–25 ml/kg/h Loading dose:2 g Maintenance dose: 1.5–1.75 g q8h (Chaijamorn et al., 2018) Dialysate rate: 30.1 ± 5.4 ml/kg/h 2 g q8h extended infusion (Philpott et al., 2019)	Combined flow rate: ≤1,000 ml/h 1 g q8h Combined flow rate: ≥1,500 ml/h 2 g q8h or 1 g q6h (Carlier et al., 2015)
**Ceftolozane/tazobactam**	Ultrafiltration rate: 20–25 ml/kg/h (pre or post-dilution), and 35 ml/kg/h (pre-dilution) Loading dose:750 mg Maintenance dose:1.5 g continuous infusion q24h Ultrafiltration rate: 35 ml/kg/h Loading dose:1.5 g Maintenance dose: 2.25 g continuous infusion q24h; Ultrafiltration rate: 20–35 ml/kg/h (pre-dilution), and 20 ml/kg/h (post-dilution) 750 mg q8h intermittent infusion Ultrafiltration rate: 25–35 m;/kg/h (post-dilution) 1.5 g q8h intermittent infusion (Chaijamorn et al., 2017)	Dialysate rate: 20–25 ml/kg/h Loading dose: 750 mg Maintenance dose:1.5 g continuous infusion q24h or 750 mg q8h intermittent infusion Dialysate rate: 35 ml/kg/h Loading dose:1.5 g Maintenance dose:2.25 g continuous infusion q24h or 1.5 g q8h intermittent infusion (Chaijamorn et al., 2017)	Combined flow rate: 1,000–1,500 ml/h Loading dose:3 g Maintenance dose: 0.75 g q8h (Sime et al., 2019) Ultrafiltration rate: 2 L/h or 1 L/h Dialysate rate: 2 L/h 3 g q8h continuous infusion (Aguilar et al., 2019; Mahmoud et al., 2020)
**Sulbactam**	Ultrafiltration rate: 30 ml/h/kg 1 g q8h (Gao et al., 2016)	Dialysate rate: 1 L/h 1g q8h (Trotman et al., 2005)	Combined flow rate: 1 L/h 1g q8h (Trotman et al., 2005)
**Meropenem**	Ultrafiltration rate: 22 ± 12 ml/kg/h 1 g q12h (MIC ≤ 1 mg/L) (Seyler et al., 2011) Ultrafiltration rate: ≥4 L/h 1 g q8h (Bilgrami et al., 2010) Ultrafiltration rate: 35 ml/kg/h 1 g q8h 1h infusion (Onichimowski et al., 2020b)	Dialysate rate: 20–35 ml/kg/h 2 g q12 h; or Loading dose:1 g, Maintenance dose:500 mg q8 h; or Loading dose:1 g LD, Maintenance dose:1 g q12 h (Shaw and Mueller, 2017) Dialysate rate:35 ml/kg/h 1 g q8h 1h infusion (Onichimowski et al., 2020b) Dialysate rate: 0.7–1 L/h 0.25 q24h for MIC ≤2 mg/L >0.5 g q8h for MIC = 16 mg/L (Kawano et al., 2015) Dialysate rate: 30 ml/kg/h 1 g q8h or 2 g q8h or 3 g continuous infusion q24h (Grensemann et al., 2020)	Ultrafiltration rate: 22 ± 12 ml/kg/h Dialysate rate: 23 ± 9 ml/kg/h 1 g q12h (MIC ≤1 mg/L) (Seyler et al., 2011) Ultrafiltration rate: 2 L/h Dialysate rate: 1 L/h 500 mg q8h (MIC ≤4mg/L) (Varghese et al., 2015) Ultrafiltration rate: 1.5–2 L/h Dialysate rate: 1–1.5 L/h 1 g q8h
**Imipenem**	Ultrafiltration rate: 52 ± 14 ml/kg/h 1.0 g q6h Ultrafiltration rate: 20 or 37 ml/kg/h 0.5 g q6h for MIC ≤2 mg/L, 1.0 g q6h for MIC 4–16 mg/L (Li and Xie, 2019)	Dialysate rate: 20 or 37 ml/kg/h 0.5 g q6h for MIC ≤2 mg/L, 1.0 g q6h for MIC 4–16 mg/L (Li and Xie, 2019)	Combined flow rate: 20 or 37 ml/kg/h 0.5 g q6h for MIC ≤2 mg/L, 1.0 g q6h for MIC 4–16 mg/L (Li and Xie, 2019)
**Panipenan**	CLtot (ml/min) = (1.2 creatinine clearance + 66.5) +0.86 (dialysate rate+ ultrafiltration rate) CLtot <80 ml/min, 0.5 q12h or 1.0 g q15h; 80 ≤ CLtot ≤120 ml/min, 0.5 q8h or1.0 g q12h; 120 ≤ CLtot ≤160 ml/min, 0.5 g q6h or1.0 g q8h (Hayakawa et al., 2006)		
**Biapenan**	–	–	Ultrafiltration rate: 1,000 ml/h Dialysate rate: 500 ml/h 300 mg q6h (Akashita et al., 2015)
**Ertapenem**	–	Dialysate rate: 1–3 L/h 500 mg q24h, 750 mg q24h, 500 mg q12h, or 1,000 mg q24h (Eyler et al., 2014)	Combined flow rate: 36–51 ml/h/kg 500 mg q24h, 750 mg q24h, 500 mg q12h, or 1,000 mg q24h (Eyler et al., 2014)
**Doripenem**	Ultrafiltration rate: 1,900–3,250 ml/h 1 g q8h (Vossen et al., 2015)	Dialysate rate: 2 L/h 1 g q8h (Vossen et al., 2015)	Combined flow rate: 1,500–2,800 ml/h 1 g q8h (Vossen et al., 2015)
**Amikacin**	Ultrafiltration rate: 30 ml/kg/h 25 mg/kg q48h (Roger et al., 2016b)	Exact dose cannot be predicted (Lam and Bauer, 2013)	Combined flow rate: 30 ml/kg/h 25 mg/kg q48h (Roger et al., 2016b)
**Levofloxacin**	Ultrafiltration rate: 14–23 ml/min 250 mg q24h (Malone et al., 2001)	Not suitable as monotherapy to treat gram-negative infections (Shaw and Mueller, 2017)	Dialysate rate: 0.8–1.5 ml/min, Ultrafiltration rate: 9–23 ml/min 250 mg q24h (Malone et al., 2001)
**Ciprofloxacin**	Ultrafiltration rate: 30 ml/kg/h 400 mg q8h (Roger et al., 2016a)	Dialysate rate: 3 L/h 200 mg q8h (Wallis et al., 2001)	Dialysate rate: 2 L/h Ultrafiltration rate: 2 L/h 400 mg q12h (MIC ≤0.5 mg/L) (Spooner et al., 2011) Ultrafiltration rate: 15 ml/kg/h dialysate rate: 15 ml/kg/h 400 mg q8h (Roger et al., 2016a)
**Polymyxin B**	No change	No change	No change
**CMS**	Ultrafiltration rate: 35 ml/kg/h Loading dose: 9 million U Maintenance dose:4.5 million U tid (Honore et al., 2014; Spapen et al., 2019)	Dialysate rate: 42 ml/min A loading CBA dose: C_ss,avg_ (mg/L) × 2.0 × body weight (kg) Maintenance dose: 192 mg × C_ss,avg_ (mg/L) q8–12h (Garonzik et al., 2011) Dialysate rate: 1–1.5 L/h Loading dose: 9 million U Maintenance dose:4.5 million U q12h (Leuppi-Taegtmeyer et al., 2019)	Ultrafiltration rate: 0.5–0.9 L/h, Dialysate rate: 1.0–1.5 L/h Loading dose: CMS 12 million U Maintenance dose: 6.5–7.5 million U q12h (Karaiskos et al., 2016)
**Clindamycin**	No change	No change	No change
**Azithromycin**	No change	No change	No change
**Tigecycline**	No change	No change	No change
**Vancomycin**	Ultrafiltration rate: 30–40 ml/kg/h 400–650 mg q12h (Li et al., 2020)	Dialysate rate: 25 ml/kg/h Loading dose: 1.5 g Maintenance dose: 500 mg q8h (van de Vijsel et al., 2010)	Loading dose: 15–20 mg/kg Maintenance dose: 500 mg (7.5–10 mg/kg) q24h (Matsumoto et al., 2013)
**Teicolanin**	Loading dose: 1,200 mg Maintenance dose: 600–1,800 mg q24h (Bellmann et al., 2010)	Dialysate rate: 16 ml/min Loading dose: 800 mg Maintenance dose: 400 mg q48–72h (Wolter et al., 1994)	–
**Daptomycin**	–	Dialysate rate >30 ml/kg/h 6 mg/kg q24h (Corti et al., 2013; Xie et al., 2020)	Combined flow rate >30 ml/kg/h 6–8 mg/kg q24h (Corti et al., 2013; Xu et al., 2017; Xie et al., 2020)
**Linezolid**	No change	No change	No change
**SMX/TMP**	Ultrafiltration rate: 1, 2, 3, 6 L/h TMP 10 mg/kg/day divided q12h (Kesner et al., 2014)	Dialysate rate: 1, 2, 3, 6 L/h TMP 10 mg/kg/day divided q12h (Kesner et al., 2014)	–
**Fluconazole**	Ultrafiltration rate: 1 L/h 400 mg q24h (Trotman et al., 2005) Ultrafiltration rate: 2.4–3.2 L/h Loading dose:12 mg/kg lean body weight Maintenance dose:6 mg/kg lean body weight q24h (Lopez and Phillips, 2014)	Dialysate flow rate: 1 L/h 800 mg q24h (Trotman et al., 2005) Dialysate flow rate: 4 L/h Loading dose: 900 mg Maintenance dose:600 mg q12h (Oualha et al., 2019)	Ultrafiltration rate: 2 L/h Dialysate flow rate: 1 L/h 400 mg q12h (Patel et al., 2011)
**Voriconazole**	No change	No change	No change
**Posaconazole**	No change	No change	No change
**Caspofungin**	No change	No change	No change
**Micafungin**	No change	No change	No change
**AMB**	No change	No change	No change
**Acyclovir**	Ultrafiltration rate: 1 L/h 5–7.5 mg/kg q24h (Trotman et al., 2005)	Dialysate flow rate: 1 L/h 5–7.5 mg/kg q24h (Trotman et al., 2005)	Ultrafiltration rate: 1 L/h Dialysate flow rate: 1 L/h 5–7.5 mg/kg q24h (Trotman et al., 2005)
**Ganciclovir**	–	Dialysate flow rate: 1 L/h 5mg/kg q48h (Bastien et al., 1994)	Ultrafiltration rate: 1 L/h Dialysate flow rate: 1 L/h 2.5 mg/kg q24h (Horvatits et al., 2014)
**Oseltamivir**	–	Dialysate flow rate: 30.8 ± 3.57 ml/kg/h less than 150 mg q12h (Eyler et al., 2012)	Ultrafiltration rate: 1.5 L/h Dialysate flow rate: 1.5 L/h less than 150 mg or 75 mg q12h (Lemaitre et al., 2012)
**Peramivir**	Ultrafiltration rate: 48 ml/kg/h 600 mg q24h (Scheetz et al., 2011)		Ultrafiltration rate: 1 L/h Dialysate flow rate: 1 L/h 600 mg q24h (Bazan et al., 2010)

CLtot, total clearance; CBA, colistin base activity.

## CRRT Factors Influencing the PK/PD of Antimicrobials

### CRRT Modalities

CRRT mainly encompasses several therapeutic modalities (Tandukar and Palevsky, 2019). Continuous venous–venous hemodialysis (CVVHD), continuous venous–venous hemofiltration (CVVH), and continuous venous-venous hemodiafiltration (CVVHDF) are the major modalities applied during therapy (Jiang et al., 2014). The modalities mentioned above involved three main techniques of clearance, including hemodialysis, hemoﬁltration, and hemodiaﬁltration (Tolwani, 2012). The clearance mechanism of hemodialysis is diffusion, which drives the solutes from a more concentrated to a less concentrated area. Drugs with small molecular weight (<500–1,000 Da) could be removed efficiently by hemodialysis. Dialysis ﬂuid is required for hemodialysis. The clearance mechanism of hemofiltration is convection, which means the solutes move through a membrane with a pressure gradient. Drugs with larger molecular weight could be efficiently cleared by hemofiltration. Replacement fluid is required for hemofiltration. Hemodiaﬁltration combines diffusion and convection mechanisms. Solutes are removed by concentration gradient and pressure gradient. Both dialysis fluid and replacement ﬂuid are required for hemodiaﬁltration. In general, drug removal may change in different CRRT methods, therefore we complied the dosing adjustment of antimicrobials basing on CRRT modalities in the present works.

### CRRT Membrane

CRRT membranes vary in permeability, membrane composition, and contact area, which lead to the differences in drug clearance. The ability of drugs to pass through a filter membrane can be represented by the sieving coefficient (SC) and the saturation coefficient (SA). The SC and the SA indicate the ratio of solute concentration in ultrafiltrate (SC) or the dialysate (SA) to solute concentration in the blood (Wong et al., 2015). CRRT clearance can be calculated by SC/SA and effluent flow rates as follows: convention clearance (ml/min) = SC × ultrafiltrate flow rate (ml/min); diffusion clearance = SA × dialysate flow rate (ml/min) (Pistolesi et al., 2019). SC or SA = 0 represents all drugs cannot pass through the membranes, while SC or SA =1 represents that all drugs can be filtered through the CRRT membrane.

Compared with intermittent hemodialysis filter membranes, CRRT membranes have increased pore size and can remove larger molecules effectively. Different CRRT membrane materials influence the clearance of antimicrobials. For example, polysulfone, polymethylmethacrylate and polyacrylonitrile membranes are typically used for CRRT. Among these membranes, polyacrylonitrile membranes have a hydrogen structure made of acrylonitrile/methallyl sulfonate copolymers, could absorb a large amount of proteins (Michikoshi et al., 2019). Compared with polysulfone, polyacrylonitrile filter membranes absorbed a large portion of the administered dose of antibiotics, such as gentamicin and tigecycline (Onichimowski et al., 2020a). Additionally, the surface area of the CRRT filters significantly increased from 0.6–0.9 to 1.2–1.5 m^2^ over the past few years (Onichimowski et al., 2020a). A recent study demonstrated that patients receiving CVVHDF with 1.5 m^2^ AN69ST membranes required higher piperacillin/tazobactam doses than 0.9 m^2^ membranes (Ulldemolins et al., 2016). The increased membrane surface area might partially account for the augment of drug absorption. Membrane characteristics during CRRT should be taken into consideration while dosing adjustment. However, due to the limited studies on the antimicrobial absorptions of CRRT membranes, accurate dose recommendations are currently not available.

### Dilution Mode and Effluent Flow Rate

Fluid can be infused before the ﬁlter (pre-dilution) or after the ﬁlter (post-dilution). In post-dilution mode, the plasm passes through the membrane directly, therefore the drug removal is associated with flow rate and sieving coefficient. In pre-dilution mode, plasm is diluted before filter leading to a decreased drug concentration before filtration, thus the clearance of drugs is relatively lower (Tandukar and Palevsky, 2019).

Effluent flow rate is the sum of dialysate and ultrafiltrate flow rates (Van Wert et al., 2010). KDIGO 2012 clinical practice guideline (Stevens and Levin, 2013) and KDOQI in 2013 (Palevsky et al., 2013) recommended that patients treated with CRRT receive an effluent flow rate of 20 to 25 ml/kg/h. However, CRRT dosing remains highly variable in practice despite the national guidelines (Tandukar and Palevsky, 2019). Several studies have demonstrated that higher flow rate increases the clearance of some drugs, and may need higher antimicrobial dose regimens (Ohchi et al., 2011; Jamal et al., 2014; Boucher et al., 2016; Chaijamorn et al., 2017; Kohama et al., 2017). Although simply adjusting doses to effluent rate is insufficient to assure reaching antibiotic exposure goals due to the variability of critically ill patients (Roberts et al., 2012; Jang et al., 2019). It is still important and necessary to consider the effluent flow rate of CRRT for antimicrobial agents dosing strategies (Roberts et al., 2015).

## Antimicrobials Dosing Strategies during CRRT

The ability of drugs removal in CRRT can be affected by their own properties. Molecular weight, protein binding, drug elimination pathways and volume of distribution (Vd) are the regarded as most important factors to affect PK parameters during CRRT. CRRT can remove larger molecule drugs than low flux hemodialysis. Most of the antimicrobial drugs have a molecular weight <750 Da, while some antimicrobials with larger molecular weight can be effectively filtered under hemofiltration and hemodiafiltration modes. Drugs bind to protein would result in the formation of large molecular compounds which are difficult to remove by CRRT. However, some studies showed that some antimicrobials with high protein binding have high SC or SA (Stevenson et al., 2008). This may due to the altered clinical conditions (e.g., blood pH, hypoproteinemia, drug competition) in critically ill patients receiving CRRT. CRRT significantly increase the clearance rates of drugs mainly eliminated by the kidney. Considering the body fluid removal, CRRT may also increase the clearance rate of drugs eliminated by other organs to some extent. Drugs with small Vd are usually hydrophilic drugs, which are generally eliminated by renal. Drugs with large Vd are usually lipophilic drugs, which are typically eliminated by other organs. These drugs are less likely to be influenced by renal replacement therapy. However, CRRT act as a continuous therapy may increase the drug redistribution from tissues to vascular, and may have greater impacts on clearance of drugs with large Vd.

Moreover, PK/PD goals are key indicators to evaluate the efficacy of drug dosage regimens during CRRT. In simple terms, antimicrobials could be classified into time-dependent antibiotics and concentration-dependent drugs. The index associated with time-dependent action is time above the MIC (%T > MIC), while the index associated with concentration-dependent action are the ratio of peak concentration and MIC (C_max_/MIC) and the ratio of 24-h area under the curve and MIC (AUC_24_/MIC) (Mueller et al., 2004). One-size-fits-all dosing in critically ill subjects receiving CRRT might be improper (Afshartous et al., 2014). However, we tried to make dosing recommendation for adult patients according to CRRT modalities and flow rates in the present work. The search strategy for the literature selection used was: (drug name) AND (continuous renal replacement therapy OR hemodialysis OR hemofiltration OR hemodiafiltration OR CVVH OR CVVHD OR CVVHDF).

### Antimicrobials Which Are Required for Dosing Adjustment

#### Flucloxacillin

Flucloxacillin is mainly eliminated by the kidney. Flucloxacillin has a low SC (mean SC 0.3) under CVVH. It could be explained by the high protein binding rate of flucloxacillin (Bouman et al., 2006). Despite of the low SC, serum levels of flucloxacillin significantly decreased after ultrafiltration. This may be related to the adsorption of polyamide membrane (Meyer et al., 2003). A flucloxacillin regimen of 4.0 g q8 h seems to be appropriate for most of the methicillin-susceptible Gram-positive infection treatment during CVVH. The recommended dose of flucloxacillin during CVVH is higher than usual dose (1–6g daily).

#### Piperacillin/Tazobactam

Both piperacillin and tazobactam are eliminated *via* the kidney by glomerular filtration and tubular secretion. Piperacillin/tazobactam can be removed in different kinds of CRRT modalities. Higher but not significant piperacillin clearance was reported in patients receiving CVVHDF compared with CVVH (Roger et al., 2017a). Meanwhile, no significant impact between CVVHD and CVVHDF on the piperacillin/tazobactam PK parameters (Seyler et al., 2011). Earlier studies indicated that 2.25 g q6h to 3.375 g q6-8h was suitable for CVVH, and 3.375 g q6h was appropriate for CVVHD and CVVHDF (Trotman et al., 2005; Heintz et al., 2009). A population pharmacokinetics study indicated that piperacillin/tazobactam 4.5 g q6h provided high probability of target attainment under CVVH mode (Asin-Prieto et al., 2014). Continuous infusion of piperacillin/tazobactam could significantly improve PK targetS attainment (Jamal et al., 2015; Shotwell et al., 2016; Roger et al., 2017a; Richter et al., 2019). A high piperacillin/tazobactam dose (18 g daily) using continuous infusion is recommended to treat less susceptible pathogens or patients with septic shock during CRRT.

#### Ceftazidime/Avibactam

Ceftazidime/avibactam is a novel β-lactam/β-lactamase inhibitor combination used in multidrug-resistant gram-negative infections. Limited data were available for ceftazidime/avibactam dosing during CRRT. Both ceftazidime and avibactam could be removed by CVVH, but the dosing strategy during CVVH was not clear (Wenzler et al., 2017). A case report found that 2.5 g infused over 2 h q8h was appropriate during CVVHDF (Soukup et al., 2019). According to the limited available evidence, a usual dose regimen is recommended, while further study is urgently needed.

#### Cefepime

About 85% of cefepime is excreted from urine as unchanged drug. Cefepime can be removed by CRRT. Earlier studies recommended 1–2 g q12h cefepime under CVVH and 1 g q8h or 2 g q12h under CVVHD or CVVHDF (Trotman et al., 2005; Heintz et al., 2009). A recent study showed that the variability in cefepime PK parameters during CVVH or CVVHDF. Monte Carlo methods indicated that 2 g q8h was suitable for high flow rate (>1.5 L/h), while 1 g q8h was appropriate for low flow rate (≤1 L/h) under CVVH or CVVHDF (Carlier et al., 2015). The optimal dosing should be adjusted based on CRRT modalities, MICs and flow rates (Chaijamorn et al., 2018). Extended infusion of cefepime improves the PK/PD target attainment (100% fT > MIC_8_) during CVVH and CVVHD (Philpott et al., 2019). Dosing of 1–2 g q8–6h are recommended during CRRT under different modalities.

#### Ceftolozane/Tazobactam

Ceftolozane/tazobactam is mainly excreted from urine. An *ex vivo* study showed that ceftolozane/tazobactam transmembrane clearances did not differ at the equivalent effluent rate in CVVH and CVVHD models, and neither polysulfone nor AN69 membranes absorb ceftolozane/tazobactam. Ceftolozane/tazobactam clearances mainly depended on effluent flow rates (Chaijamorn et al., 2017). Compared with a patient with normal renal function, a patient who receiving CVVHDF had decreased ceftolozane clearance (Bremmer et al., 2016). During CVVHDF, 0.75 g q8h in the initial 24 h could achieve a 40% fT >MIC, while doses of at least 1.5 g q8h were required for 100% fT >MIC (Sime et al., 2019). A case study showed the concentrations of ceftolozane were eight times the susceptibility breakpoint for the entire dosing interval after extended-infusion ceftolozane/tazobactam at 1.5 g q8h during CVVH (Oliver et al., 2015). Case reports showed that ceftolozane/tazobactam 3 g q8h continuous infusion could be effective and safe during CVVHDF (Kuti et al., 2016; Aguilar et al., 2019; Mahmoud et al., 2020). Dose regimens range from 1.5 to 4.5 g daily are recommended according to different effluent flow rates and CRRT modalities.

#### Sulbactam

Some 84% sulbactam were eliminated by the kidney. According to the study on Ampicillin-sulbactam, earlier study indicated sulbactam 1 g q12h under CVVH and 1 g q8h under CVVHD or CVVHDF (Trotman et al., 2005). A recent study indicated that the pharmacokinetics of sulbactam in critically ill patients were altered in critically ill patients undergoing CVVH, and a higher dose was recommended (Gao et al., 2016). All patients who receiving a sulbactam dose of 1 g q8h achieved sulbactam concentrations >8 mg/L more than 50% of the dosing interval for the initial dose, while only three out of eight achieved the trough concentrations >8 mg/L at steady stage. Higher dose regimen might be required during CVVH for less susceptible pathogens. Until now the PK/PD of sulbactam under CVVHDF and CVVHD have not been updated. Therefore, therapeutic drug monitoring (TDM) would be recommended to individualize the dose regimen.

#### Meropenem

Meropenem is eliminated by the kidney. CRRT including CVVH (Isla et al., 2008; Bilgrami et al., 2010; Seyler et al., 2011; Beumier et al., 2014; Sime et al., 2018a; Onichimowski et al., 2020b), CVVHDF (Isla et al., 2008; Seyler et al., 2011; Beumier et al., 2014; Varghese et al., 2015), and CVVHD (Afshartous et al., 2014; Kawano et al., 2015; Shaw and Mueller, 2017; Nowak-Kozka et al., 2020; Onichimowski et al., 2020b) could eliminate meropenem. A wide range of meropenem dose regimens from 0.25g q24h to 2 g q8h have been shown to be effective and were recommended for CRRT with diverse effluent flow rates (Kawano et al., 2015; Grensemann et al., 2020). The variable dose regimens are closely related to the MICs of pathogens in order to achieve the different PK/PD goals (e.g., 40% T > MIC, 100% T > MIC). Additionally, increased total clearance of meropenem was observed in patients with higher creatinine clearance (Isla et al., 2005), residual renal function should also be taken into consideration when adjusting the meropenem dose during CRRT (Ulldemolins et al., 2015). Continuous infusion of meropenem seems to be an effective method to improve the clinical efficacy for patients with less susceptible pathogen infections or augmented renal clearance during CRRT (Langgartner et al., 2008; Burger et al., 2018; Nowak-Kozka et al., 2020). Dose regimens should be adjusted according to different effluent flow rates and MICs of pathogens.

#### Imipenem

Imipenem and its metabolites were eliminated in urine. Early study indicated that 1.0 g/day imipenem achieved concentrations adequate to treat most common gram-negative pathogens (MIC up to 2 mg/L) and 2.0 g/day or more may be required to treat the pathogens with MIC = 4 to 8 mg/L during CVVH or CVVHDF (Fish et al., 2005). A recent study found that high-dose CVVH resulted in a high overall clearance of imipenem (Boucher et al., 2016). Monte Carlo simulations demonstrated that the MIC of pathogens and CRRT intensity (the sum of ultrafiltration rate and dialysis rate) were important factors for dosing strategies of imipenem (Li and Xie, 2019). In summary, the dose regimens of imipenem should be adjusted according to the MIC of pathogens and effluent flow rates.

#### Panipenem

Panipenem is primarily excreted by the kidney. A pilot study indicated that total clearance should be calculated to evaluate the dose of panipenem under CRRT. The dose of panipenem should be calculated according to creatinine clearance, dialysate flows and ultrafiltrate flows during CRRT (Hayakawa et al., 2006).

#### Biapenem

Biapenem is eliminated by the kidney. The PK/PD of biapenem was only investigated during CVVHDF. About 300 mg q8h had a higher therapeutic efficacy than q12h (Suyama et al., 2008). Early study showed that the minimum dosages to achieve T > MIC of more than 30% at filtrate-dialysate flow rates of 1.4, 2.8, and 5.6 L/h were 300 mg q12 h, 600 mg q12 h, and 600 mg q12 h, respectively (Ikawa et al., 2008). Biapenem 300 mg q6h might be an appropriate dose under continuous hemodiafiltration (ultrafiltration rate: 1,000 ml/h, dialysate rate: 500 ml/h) (Akashita et al., 2015). Both MICs of pathogens and CRRT effluent flow rates should be considered during biapenem dosing adjustment.

#### Ertapenem

Ertapenem is mainly eliminated by kidney. *In vitro* models demonstrated that ertapenem was cleared substantially under hemodialysis or hemofiltration despite of the high protein binding (Stevenson et al., 2008; Eyler et al., 2014). Ertapenem SA declined with increasing dialysate rates for AN69 hemodiafilters, while SA for polysulfone filters or SC for AN69 and polysulfone filters did not differ at any effluent rates (Stevenson et al., 2008). This effect is due to dialysate traversing more rapidly through the hemodiafilters, however the total clearance was still rising as the dialysate flow increases. Ertapenem dosing regimens (500–1,000 mg daily) could achieve ≥40%T > MIC for at least 96% of patients during CVVHD and CVVHDF (Eyler et al., 2014). Therefore, a range of ertapenem dose regimens from 0.5 g q24h to 1 g q24h are recommended for CRRT with diverse effluent flow rates.

#### Doripenem

Doripenem is eliminated by the kidney. Doripenem was not absorbed onto AN69ST, polymethylmethacrylate, and polysulfone membranes (Hiraiwa et al., 2020). CVVHD could accelerate the elimination of doripenem (Tanoue et al., 2011). The initial dosing recommendation of doripenem for renally competent patients was 500 mg q8h. Based on this regimen, several studies suggested that doripenem dose should increase to 1.0–1.5 g/day under high flow CVVHDF (Ohchi et al., 2011; Roberts et al., 2014; Tamme et al., 2015). Doripenem clearance was highly correlated with the effluent flow rate during CVVHDF, 250 mg of doripenem q12h provided adequate plasma concentrations at a low effluent flow rate (dialysis rate, 500 ml/h; hemofiltration rate, 300 ml/h) (Hidaka et al., 2010). In June 2012, the European Medicines Agency concluded that an increased dosage of 1 g q8h should be employed for renally competent patients. According to the updated dose regimen, studies found that administration of doripenem 1 g q8h during CRRT was effective and safety (Vossen et al., 2015), and doripenem 1 g q8h combined with a loading dose of 1.5 to 2 g may be appropriate for critically ill patients who receiving CVVH, CVVHD, and CVVHDF.

#### Amikacin

Amikacin is excreted from urine. A first dose of ≥25 mg/kg amikacin is required to reach therapeutic peak concentrations under CVVHDF (dialysate flow rate: 20–40 ml/kg/h and ultrafiltration rate 25–50 ml/kg/h) (Taccone et al., 2011). A strategy of an extended-interval high loading dose of amikacin (25 mg/kg every 48 h) associated with therapeutic drug monitoring should be the preferred approach for amikacin under CVVH and CVVHDF (30 ml/kg/h) (Roger et al., 2016b). Studies (Lam and Bauer, 2013) observed a wide range of C_max_ and T_1/2_ during CVVHD, and indicated that the exact amikacin dosing regimen cannot be accurately predicted based on the dialytic dose or other factors available at the bedside during CVVHD. Amikacin dose regimens should be individualized for each patient on CVVHD based on first-dose PK assessment. No accurate dose regimen has been recommended during CVVHD. Therefore, TDM is recommended to individualize the dose regimen during CRRT.

#### Ciprofloxacin

About 50–70% ciprofloxacin excretes from urine, while 14% excretes in bile and feces after intravenous administration. CVVHD could eliminate ciprofloxacin (Roehr et al., 2015). The earlier study demonstrated that ciprofloxacin 200 mg q8h might be appropriate under CVVHD with dialysate effluent 3 L/h (Wallis et al., 2001). Spooner found that 400 mg q12h may be necessary to achieve the desired PK/PD goals in patients for a MIC ≤0.5 mg/L on CVVHDF (dialysate rate: 2 L/h, ultrafiltration rate: 2 L/h) (Spooner et al., 2011). Roger et al. indicated that a high PK variability of ciprofloxacin was occurred during CVVH and CVVHDF with no significant differences in clearance. High ciprofloxacin dosing (400 mg q8h) should be used during CVVH (ultrafiltration rate: 30 ml/kg/h) or CVVHDF (ultrafiltration rate: 15 ml/kg/h, dialysate rate: 15 ml/kg/h) if TDM is not routinely available (Roger et al., 2016a). In summary, 200 mg to 400 mg q8h are recommended under diverse effluent flow rates and CRRT modalities.

#### Levofloxacin

Levofloxacin is excreted largely as unchanged drug in the urine. Early study indicated that 250mg q24h was suitable under CVVH (ultrafiltration rate: 14–23 ml/min), CVVHDF (dialysate rate: 0.8–1.5 ml/min, ultrafiltration rate 9–23 ml/min) (Malone et al., 2001). Monte Carlo simulations found that none of the recommended dosing regimens of levofloxacin (from 314 mg q48h to 750 mg LD, 500 mg q24 h) attained the probability of target attainment goal (daily average AUC/MIC ≥125 for the first 72 h of treatment) during CVVHD (dialysate rate: 25 or 30 ml/kg/L), and therefore levofloxacin should not be used as monotherapy to treat gram-negative infections in CVVHD patients (Shaw and Mueller, 2017). According to the limited study, levofloxacin 250 mg daily might be appropriate during CVVH and CVVHDF with a relative low CRRT intensity.

#### Colistin

Colistin methanesulfonate (CMS), is the prodrug of colistin. CRRT has a dramatic impact on the pharmacokinetics of CMS, and the dose requirements of CMS are greater in patients on CRRT than for patients with normal renal function (Tsuji et al., 2019). Patients undergoing CVVH can be treated with long-term colistin therapy at doses of 3–4.5 million IU tid with a high loading dose (e.g., 9 million IU) (Honore et al., 2014; Menna et al., 2018; Spapen et al., 2019). Both CMS and colistin can be efficiently cleared by CVVHD (Leuppi-Taegtmeyer et al., 2019) and CVVHDF (Li et al., 2005; Karvanen et al., 2013; Karaiskos et al., 2016), and using a loading dose helped to achieve target colistin concentration more rapidly. A loading colistin base activity (CBA) dosage of C_ss,avg_ (mg/L) × 2.0 × body weight (kg), and maintain dosage of 192 mg × C_ss,avg_ (mg/L) q8–12 h were suitable for individual patient under CVVHD (Garonzik et al., 2011). A loading dose of 12 million IU and a maintenance dose of at least 6.5–7.5 million IU were suitable for patients undergoing CVVHDF according to a predicted pharmacokinetic model, but the safety of this high loading dose needs to be assessed (Karaiskos et al., 2016). CMS or CBA dose regimens during CRRT should be managed by a high loading dose followed by maintenance dosages based on diverse CRRT modalities and intensity.

#### Vancomycin

About 80–90% vancomycin is excreted as unchanged drug in the first 24 h from urine. Vancomycin serum levels are difficult to maintain in CRRT patients (Omrani et al., 2015), and are significantly affected by CRRT intensity (Beumier et al., 2013). Early study suggested vancomycin 1 g q48h under CVVH and 1 g q24h under CVVHD and CVVHDF (Trotman et al., 2005). This is inconsistent with theoretical knowledge: since vancomycin is a relatively large molecule drug, therefore it would be cleared more efficiently by CVVH than CVVHD theoretically. The Japanese Society of Chemotherapy and the Japanese Society of Therapeutic Drug Monitoring recommended that an initial dose of 15–20 mg/kg, and doses of 500 mg (7.5–10 mg/kg) are given q24h as maintenance doses for patients under CVVHDF (Matsumoto et al., 2013). A 1.5 g loading dose followed by continuous infusion of 1–1.5 g IV over 24 h would be recommended for patients undergoing CVVHD (van de Vijsel et al., 2010). Previous studies showed quite different recommendations on vancomycin dosage under CVVH. Chaijamorn W indicated the maintenance dose of vancomycin would be 500–750 mg q12h to provide a trough concentration of 15–20 mg/L under CVVH with an ultrafiltrate flow rate of 800–1,200 ml/h (Chaijamorn et al., 2011), while Qiang Li recommended vancomycin 400–650 mg q12h under CVVH with an ultrafiltrate flow rate of 30–40 mg/kg/h (Li et al., 2020). The latest study showed a higher dose (total dose ≥2.75g/day) of vancomycin than the current recommendation (highest literature-based dosing regimen: 1.5 g/day) was needed in CRRT patients with vancomycin MIC ≥ 1 mg/L (Charoensareerat et al., 2019). Due to inconsistent dosing recommendations in the literatures, it is recommended to adjust the individual dose according to TDM.

#### Teicoplanin

Teicoplanin is mainly eliminated by the kidney. A loading dose of 1,200 mg, and maintenance doses of 600–1,800 mg q24h to achieve trough levels of 15–25 mg/L under CVVH was recommended (Bellmann et al., 2010). Considering the large molecular weight of teicoplanin, CVVHD may not eliminate teicoplanin efficiently. Early study indicated a loading dose of 800mg and maintenance dose of 400 mg q48–72h under CVVHD (Wolter et al., 1994). CVVHDF could partly eliminate the teicoplanin according to the pharmacokinetics study, no exact dose was recommended during CVVHDF (Yagasaki et al., 2003). Due to the lacking of updated information on teicoplanin during CRRT, we suggest that TDM for the teicoplanin should be performed.

#### Daptomycin

About 78% daptomycin is excreted in urine as unchanged drug. Some studies indicated that during CVVHDF or CVVHD (combined dialysate and ultrafiltration rate: 30–40 ml/kg/h), no significant accumulation occurred with a usual dose of daptomycin in patients with normal kidney function (6 mg/kg q24h) (Corti et al., 2013), and daptomycin exposure with once-daily dosing (ranged from 3 to 8 mg/kg q24h) was similar to ICU patients with normal renal function, but lower compared to healthy volunteers (Preiswerk et al., 2013). Daptomycin at 8 mg/kg q48h in patients receiving CVVHD achieved higher peak and lower trough concentrations compared with 4 mg/kg q24h, which maximize daptomycin’s concentration-dependent activity and minimize the risk of myopathy (Vilay et al., 2011). A higher dosage regimen (8 mg/kg q24h) was shown to be appropriate for patients during CVVHDF (Xu et al., 2017). However, a recent study found that 8 mg/kg q24h daptomycin has a high probability of reaching the toxicity-related concentration threshold, while 6 mg/kg q24h gave a satisfactory risk-benefit balance during CVVHDF or CVVHD (Xie et al., 2020). In summary, daptomycin 6–8 mg/kg q24h might be appropriate during CVVHDF or CVVHD, and adverse effects should be carefully monitored after application of high dose daptomycin.

#### Sulfamethoxazole (SMX)/Trimethoprim (TMP)

SMX/TMP are mainly excreted from urine. Case study indicated that both TMP and SMX are removed by CVVHDF (dialysate rate: 1.5 L/h, ultrafiltration rate: 1.5 or 2.55L/h) to a significant degree (Curkovic et al., 2010). SMX clearance in CVVHDF showed high variability, and no accurate doses were recommended during CVVHDF. Transmembrane clearance of TMP is greater than SMX. *In vitro* study found that TMP 10 mg/kg/day and the corresponded SMX dose (50 mg/kg/day) resulted in steady state TMP peak concentrations between 5 and 10 mg/L and steady state SMX peak concentrations between 100 and 200 mg/L, which were associated with efficacy against *P. jirovecii*. TMP 10 mg/kg/day divided q12h may be an appropriate initial dose to consider in patients undergoing CVVH or CVVHD (ultrafiltration/dialysate rates: 1, 2, 3, and 6 L/h) (Kesner et al., 2014).

#### Fluconazole

Some 80% of fluconazole is eliminated by urine as unchanged drug. Early studies indicated that a dose of 800 mg q24h was suitable for patients receiving CVVHD or CVVHDF, while a dose of 400 mg q24h was suitable for patients receiving CVVH (Trotman et al., 2005). The recommended dose regimens were higher during hemodialysis than hemofiltration. The recommended dose regimens have increased due to the increase of ultrafiltrate or dialysate rates. A recent study found that a higher dose regimen (loading dose: 900 mg, maintenance dose: 600 mg q12h) could reach the theoretical target of 16–32 mg/L peak and 10 mg/L trough concentrations during CVVHD at a high flow rate (Oualha et al., 2019). Monte Carlo simulations found that a dose of 400 mg q12h maximizes empirical treatment against fungal organisms with MIC up to 16 mg/L under CVVHDF (Patel et al., 2011). A case report of an obese patient showed that a fluconazole dose of 6 mg/kg lean body weight daily was able to achieve pharmacodynamic goals (AUC: MIC ≥25) during CVVH (Lopez and Phillips, 2014). The phenomenon could be explained by the low molecular weight and low protein binding of fluconazole, which make it easy to pass through the dialysis membrane through diffusion. MICs of pathogens, CRRT modalities and effluent flow rates should be considered during fluconazole dosing adjustment.

#### Acyclovir

Acyclovir is mainly secreted and excreted by renal glomerular filtration and renal tubules. It could be cleared by renal replacement therapy. Acyclovir 5–7.5 mg/kg q24h was recommended under CVVH, CVVHD and CVVHDF previously (Trotman et al., 2005). Adding acyclovir (5.5 mg/L) to the dialysate fluid during CRRT was reported to be effective in achieving therapeutic drug concentrations (Cies et al., 2015). There are no effective new studies providing the specific dose of acyclovir in different CRRT modes. Individual TDM for acyclovir is required to optimize therapy for patients undergoing CRRT (Funaki et al., 2015).

#### Ganciclovir

Ganciclovir is mainly excreted through kidney. Early study found that ganciclovir 5 mg/kg q48h was suitable during CVVHD with a dialysate flow rate 1 L/h (Bastien et al., 1994). Horvatits et al. found that ganciclovir 2.5 mg/kg q24h was appropriate undern CVVHDF (ultrafiltration rate: 1 L/h plus dialysate flow rate: 1 L/h) (Horvatits et al., 2014). However, another case report found that the concentration of ganciclovir (2.5 mg/kg q24h) was not significantly affected by CVVHDF (ultrafiltration rate: 0.2 L/h plus dialysate flow rate: 1 L/h), and the dose would be recommended as the same as the dose for patients with creatinine clearance <25 ml/min (McGloughlin et al., 2011). We speculated that the difference between the two studies may partly due the different ultrafiltration rate. Evidences are inadequate in further studies for ganciclovir during CRRT.

#### Oseltamivir

More than 99% of oseltamivir active metabolism is excreted by the kidney. A case report showed the accumulation of oseltamivir during CVVHDF (Lemaitre et al., 2010). Studies found that oseltamivir 150 mg q12h under CVVHD (Eyler et al., 2012) and 75 mg or 150 mg q12h under CVVHDF (Lemaitre et al., 2012) yielded a median oseltamivir carboxylate AUC_0–12_ considerably higher than non-critically ill patients. These studies indicated that the doses of oseltamivir should reduce during CRRT. However, no accurate data was recommended. On the other hand, the investigated dose of oseltamivir (150 mg q12h) which was recommended by WHO for severe illness (Eyler et al., 2012) is higher than the usual oseltamivir dose 75 mg q12h, therefore the recommended dose might need to be less than 75 mg q12h for non-critically ill patients.

#### Peramivir

Peramivir is excreted mainly through urine. Peramivir could be cleared by CRRT including CVVH (Scheetz et al., 2011) and CVVHDF (Bentley et al., 2014). Under CVVH, the SC of peramivir was 0.9, and drug exposure is potentially predictable based on flow rates (Scheetz et al., 2011). Meanwhile, the SA of peramivir was 1 during CVVHDF (Bentley et al., 2014). The high SC and SA of peramivir during CRRT indicated that is readily cleared by CRRT. The number of patients was small in the studies on peramivir during CRRT. According to the limited data, peramivir 600 mg q24h was suitable during CVVH or CVVHDF (Bazan et al., 2010; Scheetz et al., 2011).

### Antimicrobials Which Do Not Need Dosing Adjustment

Classically, drugs with high protein binding and predominantly non-renal elimination are not removed by CRRT. Quite a few studies investigated the PKs of drugs with these properties. Ceftriaxone elimination *via* renal and biliary routes is about equal, while ceftriaxone clearance in patients receiving CVVH was equivalent to clearance in subjects with normal renal function (Trotman et al., 2005). The estimated Ceftriaxone clearance by CVVH was about 70% of total body clearance, and Ceftriaxone could be used safely against pathogens with a MIC   ≤  2 µg/ml (Goto et al., 2016). The PK parameters of moxifloxacin during hemodialysis were similar to patients without renal impairment (Fuhrmann et al., 2004; Tokimatsu et al., 2017). A population pharmacokinetic study showed that no dose adjustment of tigecycline seems necessary in CRRT (Broeker et al., 2018). Dosage modifications are warranted in patients receiving polymyxin B which is eliminated *via* a nonrenal route under CRRT (Tsuji et al., 2019). A PK case study indicated that no supranormal posaconazole accumulation is anticipated in a patient during CVVHDF (flow rate:1 L/h) (Sime et al., 2018b). No significant influence of CVVH on the pharmacokinetics of amphotericin B (AMB) lipid complexes has been found (Bellmann et al., 2003). CVVHDF had no clinically significant effect on the pharmacokinetics of AMB following administration of AMB lipid complexes (Malone et al., 2013). A standard dose of these antimicrobials can be recommended for patients during CRRT. Although little is known about the PK parameters of clindamycin and azithromycin during different CRRT modalities, considering their non-renal elimination, studies suggested that no dose adjustment is required during CRRT (Pistolesi et al., 2019).

A systematic review found a wide variability in linezolid PK/PD parameters across critically ill septic patients with AKI treated with CRRT (Villa et al., 2016). In patients with residual renal function, 900 mg q8h provide a high probability of treatment success without compromising the safety during CVVHD or CVVHDF (flow rate: 1,031 ± 634 ml/h) (Barrasa et al., 2019). Strategies such as TDM should be applied to improve the effectiveness of linezolid therapy under CRRT (Ide et al., 2018).

Oral formulation of voriconazole does not require dose adjustment under CRRT due to the non-renal elimination of voriconazole. The parenteral formulation is solubilized in sulfobutylether-β-cyclodextrin (SBECD), which would accumulate in patients with impaired renal function. A recent study found that SBECD could be removed by CVVH effectively (Kiser et al., 2015). Standard doses of intravenous voriconazole can be used in patients undergoing CVVH without significant risk of SBECD accumulation.

Caspofungin exposure during CVVH or CVVHD was very similar to that in healthy volunteers (Weiler et al., 2013). No caspofungin dosing adjustment is necessary for patients undergoing either form of CRRT (Roger et al., 2017b). Recent studies report different proportions of echinocandins lost by filter adsorption (Vossen et al., 2017). Unlike caspofungin, micafungin is not administered in a loading dose. Thus, the concentration of micafungin on the first day might be compromised by drug loss due to adsorption in a hemofilter, especially when high-absorptive capacity membranes and medium- to high-volume convection flows are used. The proportion of drug lost in micafungin may be clinically relevant (Gonzalez et al., 2014). Further studies should be conducted focusing on echinocandins dosing on CRRT with highly absorptive membrane filters.

## Further Directions And Conclusion

The wide variation in CRRT techniques and the heterogeneity of the critically ill patients made it difficult to decide the appropriate dosing during CRRT. Here CRRT modalities and the effluent flow rates are regarded as the main differences in the therapies. Our review provides empirical dosing strategies for the antimicrobials during CRRT based on the various CRRT modes. However, some of the recommendations were based on Monte Carlo methods and some were acquired from studies with limited samples. The majority of these recommendations are regardless of residual renal function. For patients who have residual renal function, the clearance of antimicrobials which are eliminated by kidney would elevate, corresponding increases in doses may be required. Considering the patient variability in PK/PD parameters and the different CRRT settings, therapeutic drug monitoring is suggested to optimize therapy after empirical therapy.

## Author Contributions

LL summarized the CRRT factors, wrote and edit the manuscript. XLi summarized the dosing of quinolones and aminoglycoside. YX summarized the dosing of carbapenases. YC summarized the dosing of cephalosporin and penicillins. HZ summarized the dosing of triazoles. JL summarized the dosing of colistin and glycopeptides. PL summarized the dosing of antiviral agents. YB summarized the dosing of echinocandins and amphotericin B. RZ summarized the dosing of tetracyclines and macrolides. YL summarized the dosing of linezolid and daptomycin. PY summarized the drug properties during CRRT. XLu designed the revised content, reviewed and edited the manuscript. SJ designed the content, reviewed and edited the manuscript.

## Funding

This work was supported by The National Natural Science Foundation of China (No. 81671889, No. 81703578, and No. 81800594), and the Fundamental Research Funds for the Central Universities (2019QNA7032).

## Conflict of Interest

The authors declare that the research was conducted in the absence of any commercial or financial relationships that could be construed as a potential conflict of interest.
